# Circulating tumor DNA dynamics and mutational profiles as indicators in neoadjuvant chemoimmunotherapy for resectable or borderline resectable non-small cell lung cancer

**DOI:** 10.1186/s12931-026-03868-z

**Published:** 2026-08-13

**Authors:** Wenxin Jiang, Siyu Lei, Sijie Liu, Huandong Huo, Zhuoheng Lv, Chengming Liu, Haiyan Xu, Linyan Tian, Fang Wei, Huiyang Shi, Danru Zheng, Yousheng Mao, Yan Wang

**Affiliations:** 1 Department of Medical Oncology, National Cancer Center/National Clinical Research Center for Cancer/Cancer Hospital, Chinese Academy of Medical Sciences & Peking Union Medical College, Chaoyang District, Beijing, 100021 China; 2 Department of Medical Oncology, Beijing Chest Hospital, Capital Medical University & Beijing Tuberculosis and Thoracic Tumor Research Institute, Beijing, 101149 China; 3 Department of Thoracic Surgery, Beijing Aerospace General Hospital, Beijing, 100076 China; 4 Department of Thoracic Surgery, National Cancer Center/National Clinical Research Center for Cancer/Cancer Hospital, Chinese Academy of Medical Sciences & Peking Union Medical College, Chaoyang District, Beijing, 100021 China; 5 Department of Thoracic Surgery, Peking University First Hospital, Beijing, 100034 China; 6 Department of Comprehensive Medical Oncology, National Cancer Center/National Clinical Research Center for Cancer/Cancer Hospital, Chinese Academy of Medical Sciences & Peking Union Medical College, Chaoyang District, Beijing, 100021 China

**Keywords:** Non-small cell lung cancer, Circulating tumor DNA, Immunotherapy, Immune checkpoint inhibitors, Neoadjuvant therapy

## Abstract

**Background:**

Neoadjuvant chemoimmunotherapy has reshaped the treatment landscape for non-small cell lung cancer (NSCLC), yet critical gaps remain in real-time monitoring and personalized adaptation.

**Methods:**

In this observational study, patients were evaluated via imaging and multidisciplinary review for subsequent surgery or radiotherapy after neoadjuvant chemoimmunotherapy. Serial plasma samples for circulating tumor DNA (ctDNA) dynamics and mutational analyses were collected. The endpoint was event-free survival (EFS). A pognostic mutational signature was developed using the Least Absolute Shrinkage and Selection Operator (LASSO) regression.

**Results:**

Among the 138 patients, 46.4% and 52.2% underwent surgery and radiotherapy, respectively. Compared to patients with persistently positive ctDNA, the ctDNA non-shedder exhibited a numerically longer EFS (hazard ratio [HR] 0.26, 95% CI: 0.06–1.25, *p* = 0.062), and the clearance group exhibited a statistically longer EFS (HR 0.33, 95% CI: 0.11–0.97, *p* = 0.040). Patients with positive ctDNA before surgery/radiotherapy showed a shorter EFS (HR 3.38, 95% CI: 1.26–9.08, *p* = 0.010), and positive ctDNA was correlated with a numerically shorter EFS (HR 9.72, 95% CI: 0.98–95.96, *p* = 0.051) in patients assessed as stable disease (SD) of high heterogeneity in the exploratory subgroup analysis. For patients with post-C1 negative ctDNA, no EFS difference was observed between those receiving 3–4 versus 2 cycles of chemoimmunotherapy (HR 1.54, 95% CI: 0.56–4.26, *p* = 0.41). For patients with post-C1 positive ctDNA, the post-C1 quantity was associated with clearance (AUC = 0.72, 95% CI: 0.55–0.89, *p* = 0.019). After balancing clinical characteristics, LASSO-Cox regression selected four genes (SLX4, IRF4, TP53, and SOX2) to construct a prognostic signature for EFS. The AUC at 36 months was 0.75, 0.71, 0.74 in the training, validation and full cohort. The high-risk group demonstrated a shorter EFS (*p* = 0.0002).

**Conclusions:**

Longitudinal ctDNA assessment identified high-risk subgroups in neoadjuvant NSCLC, particularly by refining prognosis in heterogeneous SD populations. An exploratory gene-based mutational signature provided potential molecular risk stratification.

**Supplementary Information:**

The online version contains supplementary material available at https://doi.org/10.1186/s12931-026-03868-z.

## Introduction

Neoadjuvant chemoimmunotherapy improves pathological complete response (pCR) and major pathological response (MPR) rates in non-small cell lung cancer (NSCLC) compared to chemotherapy, leading to improvements in event-free survival (EFS) and overall survival (OS) [1–5]. The NCCN guidelines have extended the evaluation recommendation for neoadjuvant chemoimmunotherapy to stage IB–IIIB disease [6]. However, several issues remain unresolved. First, a key challenge lies in the need to predict efficacy and guide treatment decisions using non-invasive biomarkers before or early during therapy, as relying on postoperative pathological response is clinically impractical. Although programmed death-ligand 1 (PD-L1) positivity is correlated with better survival, PD-L1-negative patients may still derive benefit [7]. Furthermore, the requirement for tissue limits its utility in cases with inadequate pre-treatment samples or for dynamic monitoring. Tumor response is assessed primarily by Response Evaluation Criteria in Solid Tumors (RECIST), while discordance between radiological findings and pathological response was often observed in NSCLC [8]. Identifying patients more likely to benefit from neoadjuvant chemoimmunotherapy earlier is crucial to avoid ineffective treatments disabling surgery [9]. Second, the optimal treatment endpoint—specifically, the ideal number of neoadjuvant cycles—remains undefined. Considerable discrepancies exist across studies [10, 11], suggesting a personalized approach, rather than a uniform standard, should be adopted.

Additionally, increasing attention has been paid to borderline resectable NSCLC, in whom neoadjuvant therapy offers multiple advantages over chemoradiotherapy followed by immune checkpoint inhibitors (ICIs) consolidation. The key point is the dual chance of local treatment, which may confer a survival benefit [12]. In a retrospective study, neoadjuvant chemoimmunotherapy has yielded high pathological response and resectability rates in borderline resectable and unresectable stage III NSCLC patients [13]. The phase II MDT-bridge study offered a feasible strategy for such patients, a multidisciplinary re-evaluation after two cycles of chemoimmunotherapy expanded the range of patients eligible for resection [14]. Therefore, the eligible population for neoadjuvant therapy may be further expanded in the future. Yet, reliable biomarkers represent an unmet need for this population.

Compared with biopsy, monitoring circulating tumor DNA (ctDNA) could be a real-time biomarker for tracking tumor evolution and assessing prognosis [15, 16]. Exploratory analyses from neoadjuvant studies have demonstrated that ctDNA clearance was correlated with improved pathological response and EFS [17, 18]. Baseline ctDNA may carry prognostic significance, although current evidence remains inconsistent [19, 20].

Given the correlation between ctDNA and both pathological response and prognosis, ctDNA may function as a indicative biomarker for neoadjuvant therapy, or even as a potential treatment endpoint. However, existing evidence is limited by focusing on ctDNA qualitative analysis at a single time-point. Therefore, we conducted this cohort study to investigate, by integrating dynamic and mutational profiles, the role of ctDNA in informing the management of resectable or borderline resectable NSCLC.

## Materials and methods

### Study design

This retrospective, observational study was to evaluate the indicative impact of ctDNA dynamics and mutational profiles in resectable or borderline resectable NSCLC patients receiving neoadjuvant chemoimmunotherapy, patients diagnosed between March 2021 and July 2025 were enrolled. The study was conducted at Cancer Hospital, Chinese Academy of Medical Sciences & Peking Union Medical College, Beijing, China, and all procedures adhered to the principles of the Declaration of Helsinki [21]. This study was approved by the Ethics-Committee of Cancer Hospital, Chinese Academy of Medical Sciences (Ethics approval number: 26/082-5807), and all participants provided informed consent for the collection of residual blood samples from routine clinical care to be used for testing.

### Inclusion and exclusion criteria

Main eligible conditions included: (1) NSCLC pathological diagnosis; (2) clinical stage II-IIIB (N0-N2 only) based on the 9th edition of the American Joint Committee on Cancer staging system; (3) neoadjuvant chemoimmunotherapy; (4) resectable or borderline resectable disease; (5) at least one measurable lesion. Main exclusion conditions included: (1) severe interstitial lung disease potentially affecting surgery or radiotherapy (RT); (2) other concomitant disease affecting surgery.

### Diagnosis and treatment procedures

Eligible patients who met the inclusion and exclusion criteria were treated per the following protocol at our institution. Patients with histologically confirmed NSCLC (via bronchoscopy or lung biopsy) must undergo enhanced computed tomography (CT) plus bone scan or positron emission tomography/computed tomography (PET/CT) and enhanced brain magnetic resonance imaging (MRI) to confirm the absence of distant metastases. PD-L1 testing was performed prior to the treatment initiation unless tissue samples are insufficient, positivity was determined by a threshold of tumor proportion score (TPS) or tumor cell (TC) ≥ 1%. For lung adenocarcinoma (LUAD) patients, the neoadjuvant therapy regimen consisted of programmed death 1 (PD-1)/PD-L1 monoclonal antibody plus pemetrexed and cisplatin/carboplatin; for lung squamous cell carcinoma (LUSC) patients, the neoadjuvant regimen consisted of PD-1/PD-L1 monoclonal antibody plus taxane and cisplatin/carboplatin. Continued adjuvant/consolidate treatment after surgery or radical RT included chemoimmunotherapy and immunotherapy. After surgery or RT, follow-up would be a 3-month interval within the first 2 years, a 6-month interval from year 2 to 5, and an annual interval beyond year 5.

### Definitions of resectability and MDT process

The baseline anatomic resectability was based on a consensus of the Society of Thoracic Surgeons (STS) [22]. Resectable disease included: T1–T3 with N0, nonbulky N1, or single‑station N2, provided that complete resection was considered technically feasible. Borderline resectable disease included: T1–T3 with bulky single‑station N2 or nonbulky multi-station N2; T4 due to size or invasion with N0 or nonbulky N1; T4 satellite with N0, nonbulky N1, or single‑station N2. Bulky nodal disease was defined as mediastinal lymph nodes with short axis diameter ≥ 2.5 cm, extranodal involvement, or confluent clusters of smaller nodes per. Since no universal standard exists for resectability after neoadjuvant therapy, we employed a multidisciplinary team (MDT) discussion. Contraindications to surgery included: (1) progressive disease per RECIST v1.1; (2) persistent unresectable anatomic features (e.g., major vascular encasement without clear regression); (3) newly developed distant metastases; (4) new contraindications to surgery; (5) unacceptable perioperative risk; (6) complete resection was considered not technically feasible by thoracic surgeons; and (7) patient refusal. All patients underwent imaging re-evaluation and MDT assessment for surgery or RT after completing two cycles of neoadjuvant therapy. An optional extension of 1–2 neoadjuvant cycles was permitted based on clinical response and treatment tolerability, prior to a re-evaluation to determine the final definitive local treatment strategy. Since all ctDNA tests were performed as post-hoc analyses, the results were not reviewed by the MDT or used to guide treatment allocation. The MDT included thoracic surgeons, medical oncologists, and radiation oncologists. Thoracic surgeons assessed anatomic resectability, operative approach, and the feasibility of complete resection; medical oncologists assessed systemic disease control, treatment response, treatment-related toxicity, and systemic treatment options; and radiation oncologists assessed the appropriateness and feasibility of definitive radiotherapy or chemoradiotherapy when surgery was not recommended. Medical operability such as performance status, cardiopulmonary reserve, comorbidities, and perioperative risk was also assessed. Final treatment decisions were reached by MDT consensus.

### ctDNA process

Blood samples were collected for ctDNA detection before neoadjuvant therapy (baseline), after each neoadjuvant therapy cycle (one day before the next cycle), and prior to the surgery/RT (after neoadjuvant therapy, within one week before the scheduled surgery or radiotherapy). ctDNA testing was performed systematically in all eligible patients (except those who declined surgery before treatment initiation or had comorbidities precluding surgery) who had available plasma samples, and no patient was excluded based on clinical judgment. Peripheral blood (3–4 mL) was collected and stored in Cell-Free DNA BCT tubes (Streck, La Vista, NE, US). Comprehensive genomic profiling of 769 cancer-related genes was performed (Genecast Precision Diagnostic Co., Ltd., Wuhan, China). For baseline samples, somatic variants were identified using a pre-defined de novo calling method [23]. To ensure high-fidelity tracking, several filtering steps were applied. Variants were filtered to exclude non-hotspot mutations exhibiting a variant allele frequency (VAF) below 0.1% or *p* > 0.001. Germline and clonal hematopoiesis variants were filtered out via comparison with paired buffy coat samples: variants were excluded if the plasma-to-BC VAF ratio was ≤ 5 (for VAF ≥ 1%) or if they lacked significant enrichment in plasma (*p* > 0.01 via Fisher’s exact test for VAF < 1%). Sample was defined as positive if at least one variant was detected. Tracking of these baseline somatic variants in subsequent longitudinal samples used thresholds of *p* < 0.05 for variant positivity and *p *< 0.01 for sample positivity [24, 25]. Concentrations of ctDNA in plasma were measured in haploid genome equivalents per mL (hGE/mL), which were calculated by multiplying the mean variant allele frequency by the input concentration of cfDNA (in pg/mL) and then dividing by 3.3 (as one hGE corresponds to 3.3 pg of DNA).

### Outcomes

The endpoint was EFS (defined as the time from the initiation of neoadjuvant therapy to disease progression, recurrence, or all-cause death).

### Machine learning identified signature genes

Genes with mutation frequency *≥*2% were included in the initial screening to ensure statistical reliability. Genes with p<0.2 in univariable analysis were considered candidate prognostic factors for EFS. The Least Absolute Shrinkage and Selection Operator (LASSO)-Cox regression with 5-fold cross-validation was employed. The penalty parameter (λ) was optimized by minimizing the partial likelihood deviance, and those genes with non-zero regression coefficients were ultimately incorporated into the prognostic signature. Baseline clinical risk factors for EFS were identified by prior Cox regression analysis, with a sensitivity analysis using multiple imputation to handle missing values in the PD-L1 variable (5 imputed datasets generated via predictive mean matching and a maximum of 20 iterations). After imputation, a Cox proportional hazards regression model was fitted to each of the 5 complete datasets, and the parameter estimates were pooled using Rubin's rules. To ensure a balanced distribution, patients with complete data were divided into the training (70%) and validation (30%) cohorts using stratified random sampling based on clinical risk factors. A multivariable Cox proportional hazards model was then constructed in the training set, incorporating the genes identified via the LASSO regression. The risk scores were calculated as the linear combination of gene mutation status weighted by their corresponding regression coefficients: signature score = *∑* (gene mutation status *×* coefficient). Based on the median risk score, patients were stratified into low- and high-risk groups. Model performance was further evaluated by calculating the concordance index (C-index) for predictive accuracy, alongside time-dependent receiver operating characteristic (ROC) curve analysis, from which the area under the curve (AUC) and 95% confidence intervals (CIs) were derived. The procedures were performed using R packages Survival, Survminer, TimeROC, Glmnet, and Caret. 

### Statistical analysis

The difference between Kaplan-Meier curves was verified by log-rank test, and the hazard ratio (HR) for EFS was calculated using Cox regression models. Patients who did not suffer any EFS events were censored at the latest tumor assessment date. All subgroup analyses were exploratory without multiple comparisons. This study employed propensity score (PS) stratification to control for confounding bias. Propensity scores were calculated using standard logistic regression incorporating PD-L1 status, tumor stage, and local treatment type as covariates. Patients were stratified into three strata (low, medium, and high propensity score) based on the PS tertiles. The primary analysis used Firth’s penalized Cox proportional hazards regression model to evaluate the effect of ctDNA status on EFS, with PS strata included as covariates for adjustment. The proportional hazards assumption of the Cox model was tested using Schoenfeld residuals. A p-value > 0.05 indicated that the proportional hazards assumption was satisfied. Multiple sensitivity analyses were conducted to assess robustness, including standard Cox regression with PS strata as stratification variable, Firth’s penalized Cox regression with direct covariate adjustment, and 1:1 propensity score matching with a caliper of 0.2 followed by standard Cox regression. The optimal cut-off for ctDNA hGE/ml was identified through ROC curves. Statistical analysis in this research was conducted by software SPSS (version 26.0) and R (version 4.5.1), and a p-value < 0.05 was considered statistically significant.

## Results

### Baseline characteristics

**Fig. 1:**
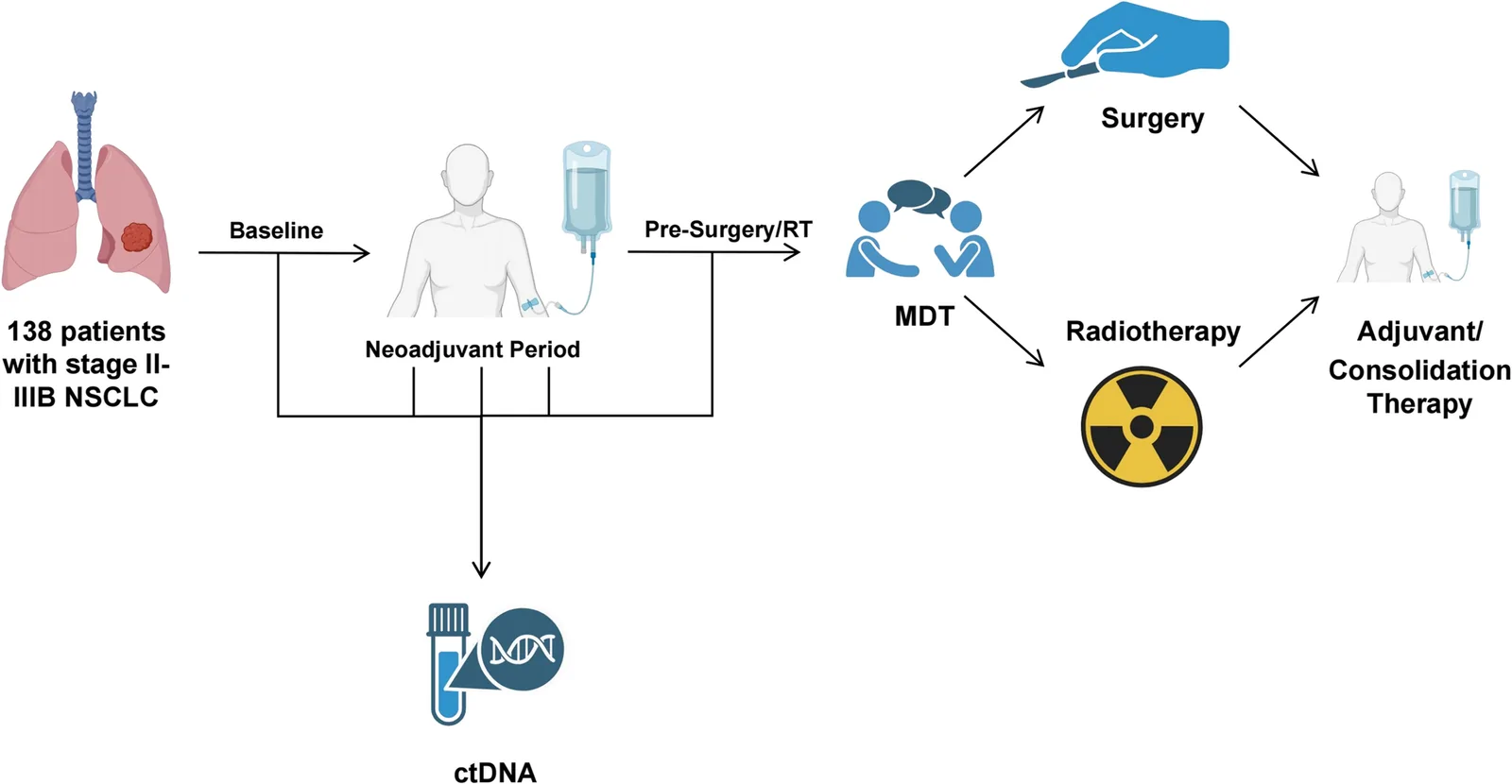
Treatment and blood sample collection procedures for patients in the cohort

Between March 2021 and July 2025, a total of 138 patients who received neoadjuvant chemoimmunotherapy were included in this study (Fig. 1; Supplementary Fig. 1). Stage IIIA was the predominant stage (50.7%), with LUSC being the most common pathological type (68.8%). PD-L1 status was assessed for the majority of patients (87.0%); it remained undetermined in cases with insufficient tissue. After neoadjuvant therapy, 46.4% and 52.2% of patients underwent surgery or definitive RT, respectively (Table 1, characteristics comparison between the two groups were shown in Supplementary Table 1).

**Table 1:** Baseline characteristics

	number
Sex, *n* (%)	
Male	119 (86.2)
Female	19 (13.8)
Age, years, median (range)	62 (22–77)
Stage, n (%)	
II	24 (17.4)
IIIA	70 (50.7)
IIIB	44 (31.9)
ECOG PS, n (%)	
0	104 (75.4)
≥ 1	34 (24.6)
Histology, n (%)	
LUSC	95 (68.8)
LUAD	36 (26.1)
Others	7 (5.1)
Smoking history, n (%)	107 (77.5)
PD-L1, n (%)	
<1%	42 (30.4)
1–49%	42 (30.4)
≥ 50%	36 (26.1)
Unknown	18 (13.0)
Subsequent treatment, n (%)	
Surgery	64 (46.4)
Radiotherapy	72 (52.2)
Systemic treatment	2 (1.5)

*ECOG PS* Eastern Cooperative Oncology Group Performance Status, *LUSC *Lung squamous cell carcinoma, *LUAD* Lung adenocarcinoma, *PD-L1* Programmed death-ligand 1

### ctDNA dynamics profile of the included patients

**Fig. 2:**
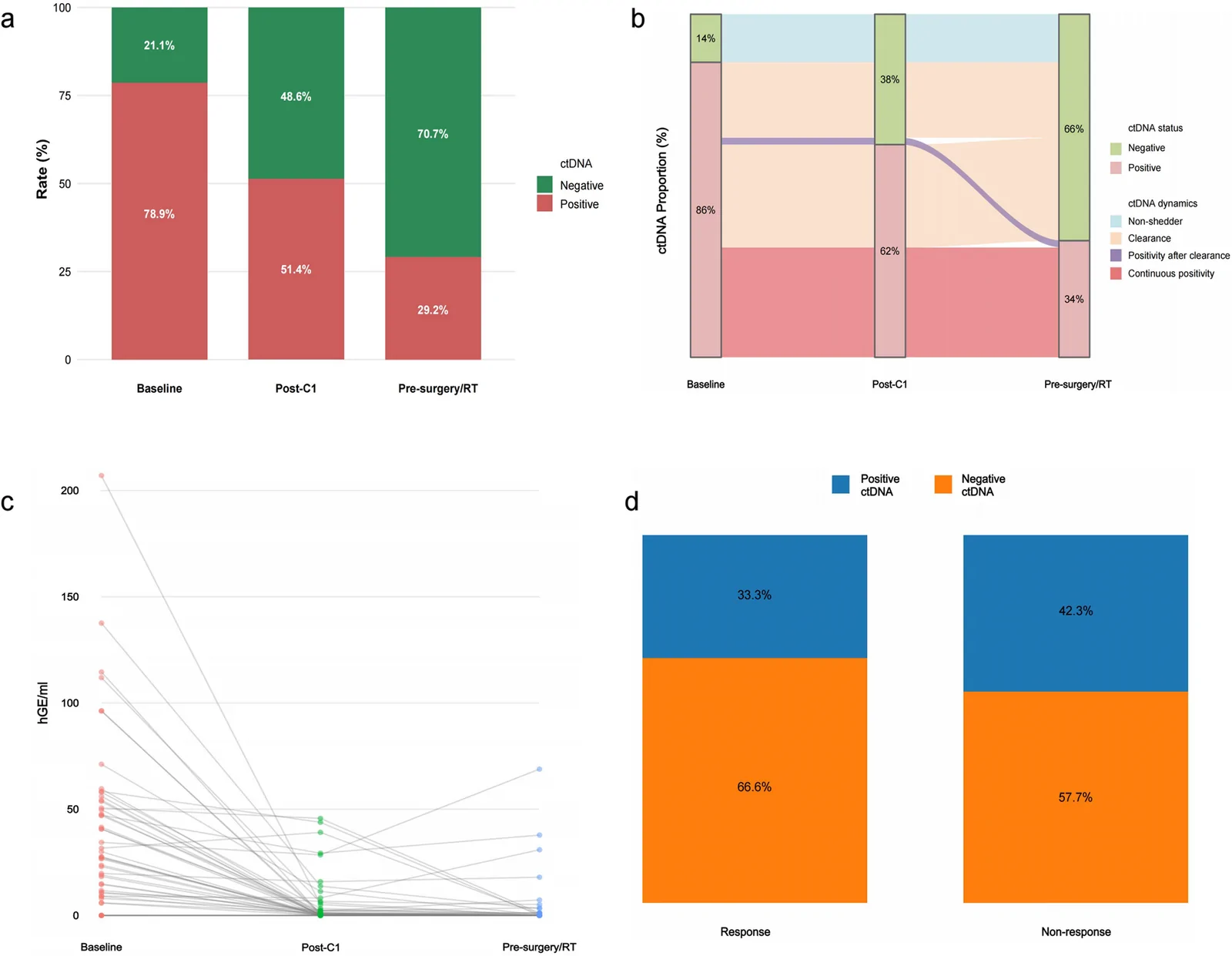
ctDNA dynamic profile of the included patients. **a** Barplot of ctDNA status for all accessible samples. **b** Sankey diagram of ctDNA status in patients with paired blood samples at baseline, post-C1, and pre-surgery/RT time points. **c** Line chart of ctDNA quantification in patients with paired blood samples at baseline, post-C1, and pre-surgery/RT time points. **d** Barplot of ctDNA status grouped by radiological response

Among patients with available ctDNA results, the positive ctDNA rates were 78.9% (105/133), 51.4% (56/109), and 29.2% (19/65) at baseline, post-C1, and pre-surgery/RT time points, respectively (Fig. 2a). Paired samples at these three time points were available from a total of 50 patients. Among patients with paired samples at three time points, 14.0% were non-shedders (defined as consistently negative ctDNA status throughout the course), 52.0% achieved ctDNA clearance (defined as baseline ctDNA positivity turning negativity before surgery/RT), and 32.0% had persistently positive ctDNA. Only one patient (2.0%) became positive again before surgery after the ctDNA clearance after C1 (Fig. 2b). The hGE/ml of the post-C1 time point significantly decreased relative to the baseline level (paired samples t-test *p* < 0.001), but the value of the pre-surgery/RT time point did not show a statistical decrease relative to the post-C1 time point (*p* = 0.294) (Fig. 2c). For patients with baseline ctDNA positivity, radiological response did not show an association with ctDNA clearance (*p* = 0.514) (Fig. 2d).

### The prognosis effect of pre-surgery/RT ctDNA in the neoadjuvant period

**Fig. 3:**
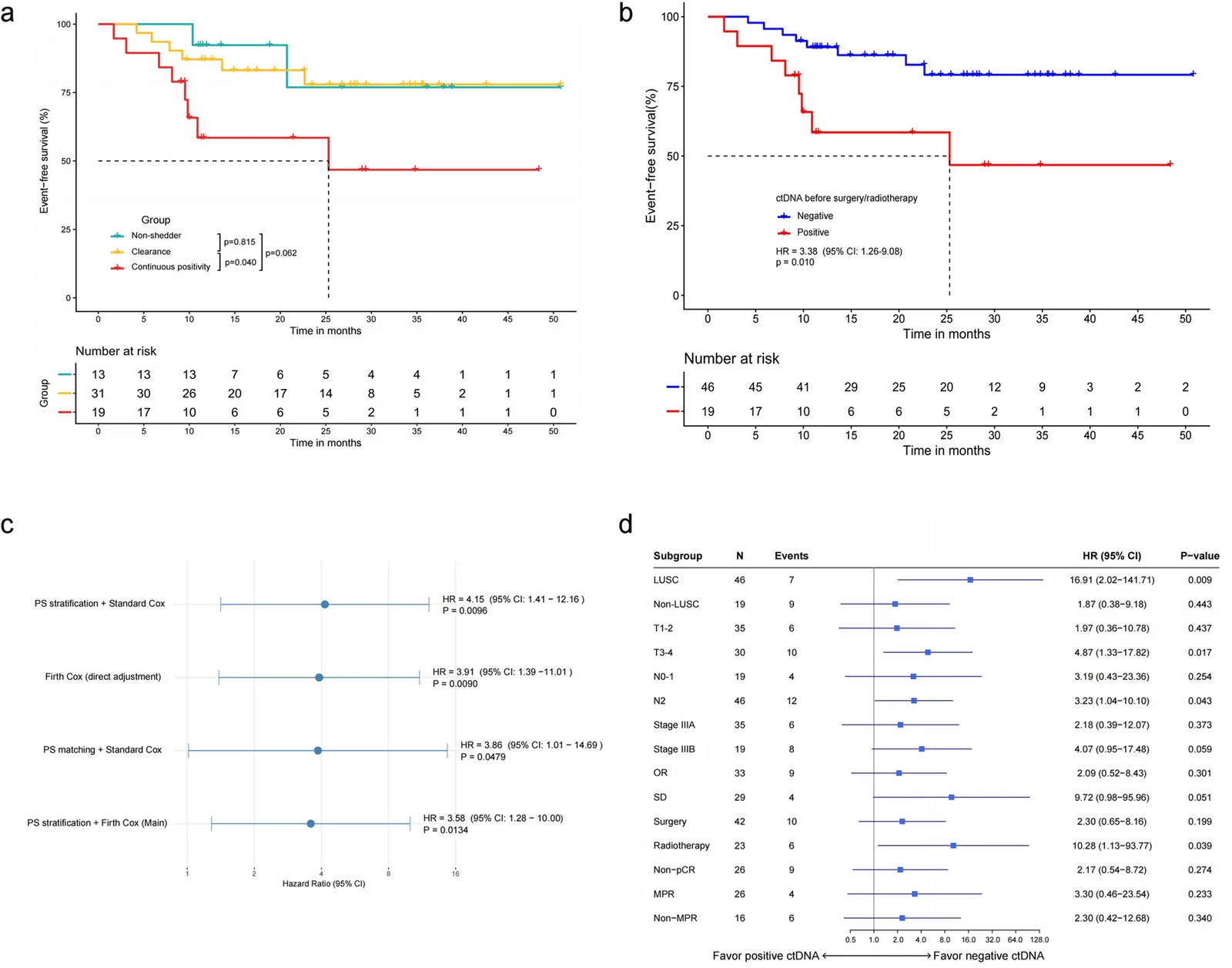
Prognostic significance of ctDNA status at the pre-surgery/RT time point. **a **Kaplan-Meier curves stratified by ctDNA dynamics in patients with paired blood samples at baseline and pre-surgery/RT time points. **b** Kaplan-Meier curves stratified by the pre-surgery/RT ctDNA status. **c** Forest plot of the effect of ctDNA positivity on event-free survival. **d** Prognostic significance of pre-surgery/RT ctDNA in different patient subgroups. LUSC: lung squamous cell carcinoma; OR: overall response; SD: stable disease; pCR: pathological complete response; MPR: major pathological response; PS: propensity score; RT: radiotherapy

As of January 31, 2026, a total of 45 EFS events occurred. With a median follow-up duration of 26.8 months (95% CI: 24.5–29.1), the median EFS of the entire cohort was not reached yet. For patients with baseline and pre-surgery/RT samples, compared to patients with persistently positive ctDNA, the ctDNA non-shedder exhibited a numerically but not statistically longer EFS (HR 0.26, 95% CI: 0.06–1.25, p = 0.062), and the clearance group exhibited a statistically longer EFS (HR 0.33, 95% CI: 0.11–0.97, p = 0.040) (Fig. 3a). Patients with positive ctDNA before surgery/RT showed a shorter EFS compared to those with negative ctDNA (HR 3.38, 95% CI: 1.26–9.08, p = 0.010) (Fig. 3b). Since the proportion of patients undergoing surgery was higher in the group with all paired samples than in the group with missing samples from any time point (Supplementary Table 2), we performed PS stratification analysis to reduce the confounding bias, an overlap of PS distributions was observed between the ctDNA-positive and ctDNA-negative groups (overlap percentage: 84.9%, Kolmogorov-Smirnov test p = 0.566) (Supplementary Fig. 2), and the proportional hazards assumption was satisfied (p = 0.781). Patients with positive ctDNA before surgery/RT showed a shorter EFS compared to those with negative ctDNA via PS stratification combined with Firth Cox regression analysis (HR 3.58, 95% CI: 1.28-10.00, p = 0.013), and the sensitivity analysis showed consistent results (Fig. 3c).

Exploratory subgroup analysis without multiple testing indicated that the potential negative prognostic significance of ctDNA presence before surgery/RT was primarily observed in patients with LUSC, T3-4 stage, N2 nodal status, and those who subsequently received radical RT (Fig. 3d), underscoring the necessity of ctDNA testing in these specific patient populations. Importantly, for the highly heterogeneous group of patients assessed as stable disease (SD) by imaging evaluation, ctDNA demonstrated potential to prognostically stratify (HR 9.72, 95% CI: 0.98–95.96, p = 0.051, Fig. 3d).

### The indicative impact of the post-C1 ctDNA in the neoadjuvant period

**Fig. 4:**
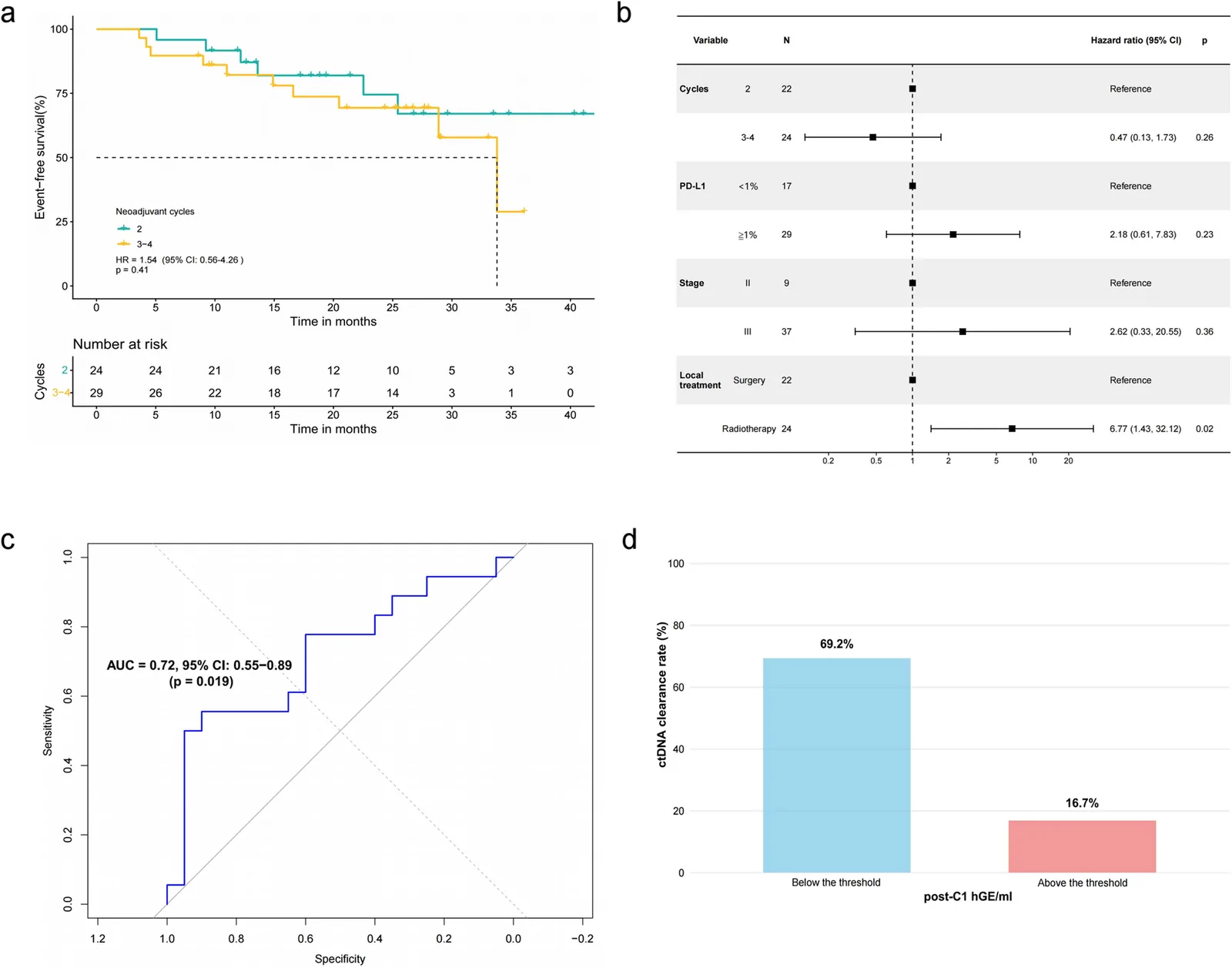
Clinical implications of post-C1 ctDNA status for neoadjuvant therapy. **a**: Kaplan-Meier curves for post-C1 ctDNA-negative patients stratified by total cycles of neoadjuvant therapy. **b**: Multivariable Firth Cox regression for EFS in post-C1 ctDNA-negative patients. **c**: ROC curve for post-C1 ctDNA quantification (hGE/ml) in indicating eventual ctDNA clearance. **d**: Eventual ctDNA clearance rate stratified by post-C1 ctDNA quantification (hGE/ml) threshold

A total of 53 patients had ctDNA-negative results after C1 treatment, while no significant EFS difference was observed between those receiving 3–4 versus 2 cycles of chemoimmunotherapy (HR 1.54, 95% CI: 0.56–4.26, *p* = 0.41; Firth Cox regression HR 0.47, 95% CI: 0.13–1.73, *p* = 0.26, patients with unknown PD-L1 status were excluded) (Fig. 4a-b).

Among 56 patients who remained ctDNA-positive after C1 treatment, ctDNA clearance was achieved in 16 patients after C2, while only 4 patients achieved clearance after C3/C4. The ctDNA hGE/ml after C1 treatment was associated with ctDNA clearance before surgery/RT (AUC = 0.72, 95% CI: 0.55–0.89, *p* = 0.019) (Fig. 4c). An optimal cut-off value was determined using the ROC curve, the group with post-C1 hGE/ml below the cut-off threshold exhibited a significantly higher final ctDNA clearance rate than that in the group above the threshold (odds ratio [OR] = 11.25, 95% CI: 1.687-119.911; Fisher’s exact test *p* = 0.005) (Fig. 4d). In contrast, baseline ctDNA hGE/ml value demonstrated no significant correlation with eventual clearance (AUC = 0.537, *p* = 0.618).

### ctDNA mutation profile and gene signature

**Fig. 5:**
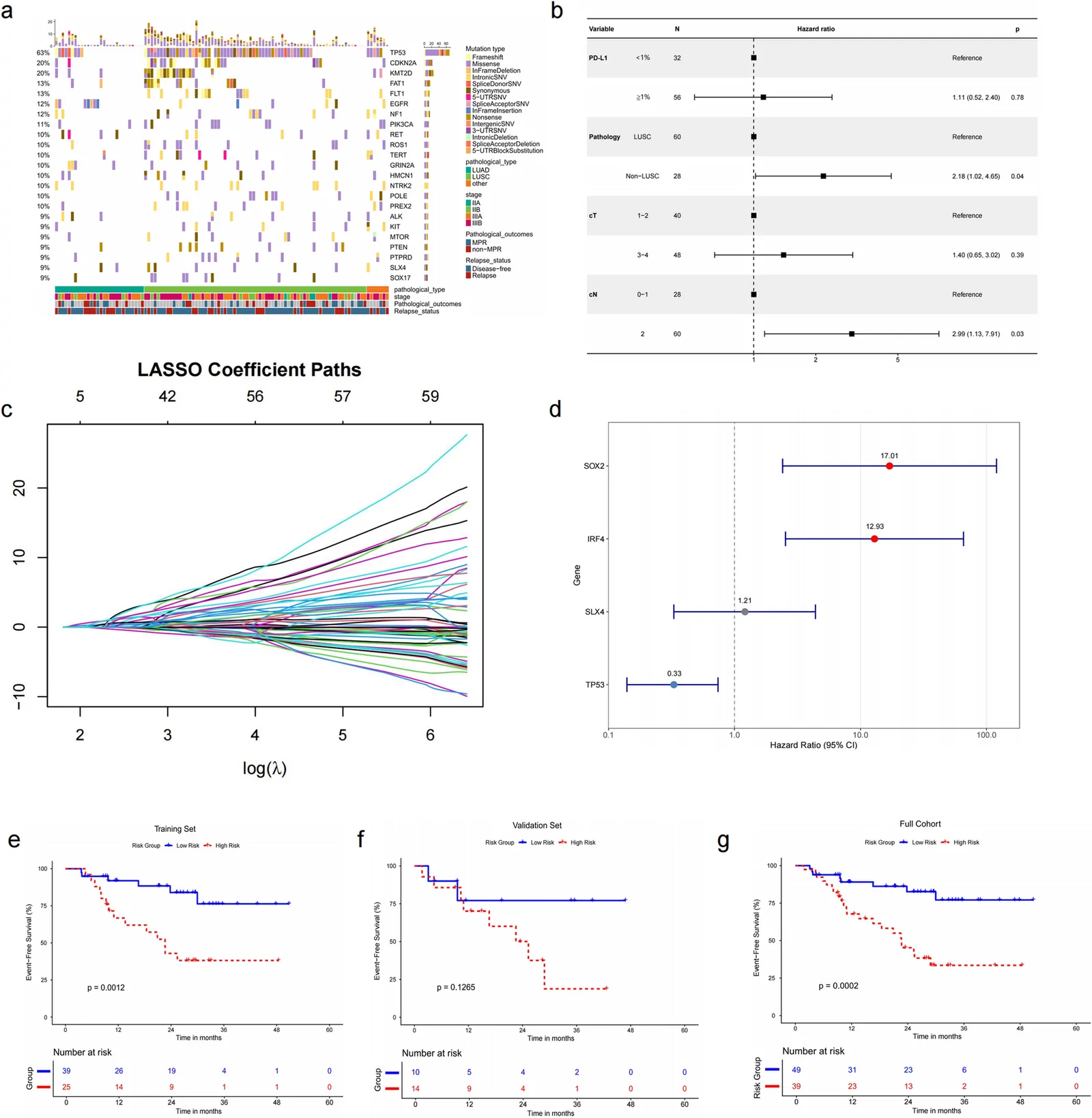
Baseline ctDNA mutation gene signature for EFS constructed via LASSO regression. **a**: Waterfall plot of baseline ctDNA-positive mutation profiles (mutation frequency >8%). **b**: Multivariable Cox regression analysis of baseline clinical characteristics. **c**: LASSO coefficient path plot for mutated genes in ctDNA. **d**: Hazard ratios of signature genes for EFS. **e**-**g**: Kaplan-Meier curves for the training (**E**), validation (**F**), and full (**G**) cohorts stratified by gene signature risk score

A total of 105 patients had baseline plasma samples available for ctDNA mutational profiling, from which a total of 1,113 single nucleotide variants (SNVs) were detected. The most frequently mutated genes included TP53 (63%), CDKN2A (20%), KMT2D (20%), FAT1 (13%), and FLT1 (13%) (Fig. 5a). Plasma common EGFR mutations were detected in 3 patients, and KRAS mutations were detected in 7 patients. Since some patients do not derive significant benefit from neoadjuvant therapy, it is necessary to explore prognostic factors prior to the initiation of neoadjuvant chemoimmunotherapy, regardless of the specific local treatment subsequently. We first performed a multivariable Cox regression analysis of baseline clinical characteristics, and the results indicated that non-squamous pathology (HR 2.18, 95% CI: 1.02–4.65, p = 0.04) and N2 lymph node metastasis (HR 2.99, 95% CI: 1.13–7.91, p = 0.03) were statistically associated with a shorter EFS (patients with unknown PD-L1 status were excluded, the sensitivity analysis of PD-L1 using multiple imputation demonstrated consistent impacts on EFS, as shown in the Supplementary Table 3) (Fig. 5b).

Next, using the training and validation sets, which were well balanced based on the pathology and N2 lymph node status (*Supplementary Table 4*), we constructed a gene signature for EFS via LASSO regression in the aforementioned 88 patients with known PD-L1 status. After a filtering described in the Methods section, LASSO-Cox regression selected four genes (SLX4, IRF4, TP53, and SOX2) with non-zero coefficients to construct the prognostic signature (Fig. 5c). The mutation frequencies of SLX4, IRF4, TP53, and SOX2 were 8.6% (9/105), 2.9% (3/105), 67.6% (71/105), and 2.9% (3/105), respectively. Multivariable Cox regression analysis in the training set demonstrated that IRF4 (HR 12.93, 95% CI: 2.54–65.73, *p* = 0.002) and SOX2 (HR 17.01, 95% CI: 2.41-120.16, *p* = 0.005) were significant risk factors for EFS, while TP53 (HR 0.33, 95% CI: 0.14–0.74, *p* = 0.007) emerged as a protective factor for EFS (Fig. 5d). Then, the risk score was calculated using the formula derived from the multivariable Cox model as described in the Methods section, the 4-gene signature exhibited a good prognostic performance, with an EFS C-index of 0.709 (95% CI: 0.615–0.803). Time-dependent ROC curves demonstrated that the gene signature achieved AUC at 24 months of 0.74, 0.64, and 0.72 in the training set, validation set, and full cohort, while AUC at 36 months was 0.75, 0.71, and 0.74, respectively (*Supplementary Fig. 3a-c*). Using the median risk score, patients were then classified into low-risk and high-risk groups. A significant difference in EFS between low-risk and high-risk groups was identified in the training set (log-rank *p* = 0.0012) and the entire cohort (*p* = 0.0002) (Fig. 5e, g). However, in the validation set, the difference in EFS did not reach the statistical significance (*p* = 0.1265, Fig. 5f), which may be attributed to the relatively limited sample size. The 24-month EFS rates in the entire cohort were 82.6% for the low-risk group and 45.3% for the high-risk group. The 36-month EFS rates were 77.1% for the low-risk group and 33.5% for the high-risk group, demonstrating an effective risk stratification by the 4-gene signature. Although the signature provided a potential risk stratification, due to the risk of overfitting caused by the lack of external validation and some low-frequency mutations, it should be interpreted as an exploratory finding.

## Discussion

In this research, tumor-agnostic ctDNA demonstrated a prognostic discrimination in resectable or borderline resectable NSCLC patients undergoing neoadjuvant chemoimmunotherapy. Analysis of ctDNA dynamics revealed the potential application for individualized treatment cycles. The 4-gene prognostic signature developed in this study demonstrated a risk stratification ability, with a C-index of 0.709 and 36-month AUC of 0.74. A key finding is the prognostic refinement within the SD population, in whom ctDNA status showed numerical differences in EFS, likely due to the limited sample size in the exploratory subgroup analysis, thereby offering a potentially actionable biomarker in settings where conventional imaging has limitations. This finding addresses a critical gap in neoadjuvant response assessment and underscores the potential of ctDNA to complement traditional imaging-based evaluation. Considering that our study included two cohorts with relative heterogeneity, namely patients with baseline resectable disease who received conventional neoadjuvant therapy and those with baseline borderline resectable disease who received broad-spectrum neoadjuvant therapy, future research should consider larger-scale validation within each single population.

While the sample size is relatively limited, our results raise the possibility that the prevailing approach to neoadjuvant duration may warrant re-evaluation, particularly in the context of ctDNA-guided response assessment. In our study, no significant EFS difference was observed between patients who received a total of 2 versus 3–4 cycles of neoadjuvant chemoimmunotherapy after achieving ctDNA clearance with C1 neoadjuvant treatment. This finding provided a novel insight into the duration of neoadjuvant therapy. However, this is inconsistent with previous studies based on smaller sample sizes [26]. Therefore, prospective large-sample studies are still needed to validate this conclusion. Currently, several studies are exploring ctDNA clearance as an endpoint for neoadjuvant therapy (such as REAL-NADIM, PRIORITY, and NCT04638582 trials), with some even proposing the possibility of omitting surgery in such patients (such as SAVED LUNG trial) [27]. Although these studies are promising, these findings should be interpreted with caution given the inherent limitations of ctDNA, including the sensitivity [28, 29], the technologies [30, 31], and the financial burden [32]. The testing platforms and methods contributing to the heterogeneity in the predictive efficacy of ctDNA must be taken into consideration. So far, two major strategies, tumor-agnostic (tumor-naive) and tumor-informed assays, have distinct advantages and limitations [33], but no consensus has been reached across studies. Although tumor-agnostic ctDNA is more suitable for the needs of dynamic monitoring before surgery, it may also lead to lower sensitivity. However, a critical distinction must be established: the sensitivity requirements for ctDNA detection differ fundamentally between on-treatment monitoring and post-surgical minimal residual disease (MRD) detection. In the post-surgical MRD setting, detecting signal at ultra-low concentrations demands the highest possible assay sensitivity, favoring tissue-informed personalized panels to track patient-specific somatic variants [34]. Our study examined on-treatment ctDNA monitoring during neoadjuvant therapy, a context in which the tumor remains biologically active and in situ, and ctDNA is shed at substantially higher concentrations. In multiple phase III trials of NSCLC neoadjuvant therapy, pretreatment ctDNA positivity rates were 83.2–89.6% using tumor-informed strategies [35, 36], which appears to be slightly higher than the baseline detection rate (78.9%) observed in our cohort using a tumor-agnostic approach. Therefore, this more clinically practical method still requires further validation, and the non-shedder group should be interpreted with caution, which may reflect technical limitations rather than actual absence of ctDNA. To address this limitation, emerging multimodal strategies, such as incorporating methylation profiling or fragmentomics, may further improve ctDNA detection in low-shedding tumor subsets [37]. Although clonal hematopoiesis of indeterminate potential (CHIP)-derived variants were filtered via paired buffy coat comparison, residual CHIP contamination cannot be entirely excluded. Unfiltered CHIP variants could elevate apparent ctDNA levels near the limit of detection or, where CHIP and tumor-tracked mutations overlap, conflate haematopoietic and tumor-derived VAF contributions, potentially resulting in false ctDNA-positive calls or overestimated ctDNA concentrations. Future studies incorporating longitudinal white blood cell sequencing and VAF kinetic modelling would further strengthen CHIP discrimination in tumor agnostic ctDNA assays. Furthermore, the impact of driver-specific ctDNA in driver oncogene-positive NSCLC should be considered. Given that our cohort comprised patients treated with chemoimmunotherapy, patients harboring driver oncogenes other than KRAS were generally excluded (unless their driver oncogene status remained unclear owing to insufficient pre-treatment tissue samples and refusal to undergo blood-based genetic testing). Although EFS is an appropriate and widely accepted endpoint in neoadjuvant trials, non-oncologic events may influence ctDNA-EFS associations. To address this, future studies could consider complementary endpoints such as landmark EFS, ctDNA-free survival, or time-dependent ctDNA modeling. These approaches can better align ctDNA dynamics with outcome assessment.

Beyond qualitative analysis of clearance, ctDNA can also provide quantitative and mutational information related to prognosis. Our research highlighted the significance of quantitative ctDNA levels after C1 neoadjuvant treatment, patients with higher C1 ctDNA levels were less likely to achieve clearance. This underscores the value of ctDNA testing at this time point, yet raises the corresponding question: should such patients consider switching neoadjuvant treatments or proceeding directly to surgery? At present, this remains unclear. Through machine learning, we constructed a genetic signature with several ctDNA mutations associated with the efficacy of neoadjuvant therapy. From our molecular perspective, the plasma interferon regulatory factor 4 (IRF4) mutation was associated with worse EFS. As a key regulator of immune responses, IRF4 is involved in the function and differentiation of both effector and immunosuppressive cells [38], and its expression elevation was correlated with a better prognosis in NSCLC patients receiving immunotherapy [39]. Modulating IRF4 represents a therapeutic strategy to reverse T-cell exhaustion and promote anti-tumor immunity. Another risk mutated gene, sex-determining region Y-box 2 (SOX2), can enhance the stem-like properties of tumor cells and contributes to the cancer stem cell population expansion [40–41]. NSCLC cell-intrinsic SOX2 signaling induces the exclusion of cytotoxic T-cells from the tumor core and fosters resistance to checkpoint blockade [42]. The Fanconi anemia pathway maintains genome stability via components including SLX4 to repair DNA interstrand crosslinks and cleave replication intermediates. However, the pathological pathway activation in cancer drives chromothripsis and generates complex genomic rearrangements and extrachromosomal DNA harboring drug-resistance genes, thereby fueling cancer genome evolution and acquired resistance [43]. Although some conflicting results exist, studies suggested TP53 as a prognostic factor for NSCLC patients receiving immunotherapy [44, 45]. Plasma TP53 mutations were associated with an improved EFS, which may be attributed to the high proportion of LUSC in our cohort, as TP53 may have different implications for immunotherapy in LUSC [46, 47]. Therefore, caution is needed when extrapolating the use of the gene signature to the broader NSCLC population. Due to the lack of validation experiments, the functional impact of these plasma mutations remains unclear, warranting further investigation.

We must acknowledge that this study had certain limitations. First, as a retrospective study, a potential source of selection bias in this analysis arises from the fact that ctDNA results were missing for some patients at specific time points. The widespread clinical application of ctDNA dynamics should be based on prospective clinical trials. Second, considering the technical variability in ctDNA, the conclusions should be validated on other standardized platforms. Third, the predominance of LUSC may restrict the generalizability of our conclusions to other NSCLC subtypes.

## Conclusion

Dynamic ctDNA identified poor-prognosis patients in neoadjuvant NSCLC, notably within heterogeneous SD populations, while ctDNA levels after C1 may indicate the clearance. A machine learning-derived mutation signature further defined the high-risk groups at the molecular prospective.

## Supplementary Information

Supplementary Material 1.

## Data Availability

The research data are available from the corresponding author upon reasonable request.

## Abbreviations

NSCLC: Non-small cell lung cancer
ctDNA: Circulating tumor DNA
EFS: Event-free survival
HR: Hazard ratio
SD: Stable disease
hGE/ml: Haploid genome equivalents per milliliter
pCR: Pathological complete response
MPR: Major pathological response
OS: Overall survival
PD-L1: Programmed death‑ligand 1
RECIST: Response Evaluation Criteria in Solid Tumors
ICIs: Immune checkpoint inhibitors
RT: Radiotherapy
cfDNA: Circulating free DNA
CT: Computed tomography
PET/CT: Positron emission tomography/computed tomography
MRI: Magnetic resonance imaging
TPS: Tumor proportion score
TC: Tumor cell
LUAD: Lung adenocarcinoma
PD-1: Programmed death 1
LUSC: Lung squamous cell carcinoma
STS: Society of Thoracic Surgeons
MDT: Multidisciplinary team
VAF: Variant allele frequency
LASSO: Least Absolute Shrinkage and Selection Operator
C-index: Concordance index
ROC: Receiver operating characteristic
AUC: Area under the curve
CIs: Confidence intervals
PS: Propensity score
ECOG PS: Eastern Cooperative Oncology Group Performance Status
OR: Odds ratio
SNVs: Single nucleotide variants
MRD: Minimal residual disease
CHIP: Clonal hematopoiesis of indeterminate potential
IRF4: Interferon regulatory factor 4
SOX2: Sex-determining region Y-box 2
