# Supplementary material for: Circulating tumor DNA dynamics and mutational profiles as indicators in neoadjuvant chemoimmunotherapy for resectable or borderline resectable non-small cell lung cancer
Source: Respir Res. 2026 Aug 13;27:438. doi: 10.1186/s12931-026-03868-z (PMC13749763; doi:10.1186/s12931-026-03868-z)
Supplement: Supplementary file 1 [file 12931_2026_3868_MOESM1_ESM.docx]

***Supplementary Fig. 1*: Screening process of the cohort**

***Supplementary Fig. 2*: Propensity score distribution by ctDNA status.**

***Supplementary Fig. 3*: Time-dependent ROC curves of the gene signature for EFS prediction in the training set (A), validation set (B), and full cohort (C)**

***Supplementary Table 1*: Comparison of clinical characteristics between patients receiving surgery and radiotherapy**

|  | Surgery (n=64) | Radiotherapy (n=72) | P-value |
| --- | --- | --- | --- |
| Sex, n (%) |  |  | 0.074 |
| Male | 52 (81.3) | 66 (91.7) |  |
| Female | 12 (18.8) | 6 (8.3) |  |
| Age, years, median (range) | 62 (22-76) | 62 (47-77) | 0.379 |
| Stage, n (%) |  |  | 0.442 |
| II | 13 (20.3) | 11 (15.3) |  |
| III | 51 (79.7) | 61 (84.7) |  |
| ECOG PS, n (%) |  |  | 0.311 |
| 0 | 51 (79.7) | 52 (72.2) |  |
| ≥1 | 13 (20.3) | 20 (27.8) |  |
| Histology, n (%) |  |  | 0.311 |
| LUSC | 42 (65.6) | 53 (73.6) |  |
| Non-LUSC | 22 (34.4) | 19 (26.4) |  |
| Smoking history, n (%) | 46 (71.9) | 60 (83.3) | 0.108 |
| PD-L1, n (%) |  |  |  |
| ＜1% | 25 (39.1) | 17 (23.6) | 0.052 |
| ≥1% | 34 (53.1) | 42 (58.3) | 0.541 |
| Unknown | 5 (7.8) | 13 (18.1) | 0.079 |

ECOG, Eastern Cooperative Oncology Group Performance Status; LUSC, lung squamous cell carcinoma; PD-L1, programmed death-ligand 1

***Supplementary Table 2*: Comparison of clinical characteristics between patients with complete paired samples and those with missing samples at any time point**

|  | Patients with all paired samples (n=50) | Patients missing samples from any time point (n=88) | P-value |
| --- | --- | --- | --- |
| Sex, n (%) |  |  | 0.566 |
| Male | 42 (84.0) | 77 (87.5) |  |
| Female | 8 (16.0) | 11 (12.5) |  |
| Age, years, median (range) | 62 (22-77) | 62 (43-76) | 0.869 |
| Stage, n (%) |  |  | 0.887 |
| II | 9 (18.0) | 15 (17.0) |  |
| III | 41 (82.0) | 73 (83.0) |  |
| ECOG PS, n (%) |  |  | 0.270 |
| 0 | 35 (70.0) | 69 (78.4) |  |
| ≥1 | 15 (30.0) | 19 (21.6) |  |
| Histology, n (%) |  |  | 0.825 |
| LUSC | 35 (70.0) | 60 (68.2) |  |
| Non-LUSC | 15 (30.0) | 28 (31.8) |  |
| Smoking history, n (%) | 41 (82.0) | 66 (75.0) | 0.344 |
| PD-L1, n (%) |  |  |  |
| ＜1% | 17 (34.0) | 25 (28.4) | 0.493 |
| ≥1% | 29 (58.0) | 49 (55.7) | 0.792 |
| Unknown | 4 (8.0) | 14 (15.9) | 0.185 |
| Surgery, n (%) | 30 (60.0) | 34 (38.6) | 0.016 |
| Median EFS, months | Not reached | Not reached | 0.337 |

***Supplementary Table 3*: Multivariable Cox regression analysis of baseline clinical characteristics in patients with baseline positive ctDNA (sensitivity analysis of PD-L1 using multiple imputation)**

| Variable | Subgroup | HR (95% CI) | P-Value |
| --- | --- | --- | --- |
| PD-L1 | ≥1% | 1.08 (0.53-2.19) | 0.841 |
|  | <1% | Reference |  |
| N stage | 2 | 2.39 (1.04-5.49) | 0.040 |
|  | 0-1 | Reference |  |
| T stage | 3-4 | 1.29 (0.64-2.58) | 0.478 |
|  | 1-2 | Reference |  |
| Pathology | Non-LUSC | 2.08 (1.05-4.12) | 0.035 |
|  | LUSC | Reference |  |

***Supplementary Table 4*: Balance of baseline clinical risk variables between the training and validation sets**

|  | Non-LUSC (n/%) | N2 (n/%) |
| --- | --- | --- |
| Training Set | 21 (32.8) | 43 (67.2) |
| Validation Set | 7 (29.2) | 17 (70.8) |
| P-value | 0.744 | 0.744 |
